# Impact of Referral Sources and Waiting Times on the Failure to Quit Smoking: One-Year Follow-Up of an Italian Cohort Admitted to a Smoking Cessation Service

**DOI:** 10.3390/ijerph15061234

**Published:** 2018-06-11

**Authors:** Lucia Borsari, Simone Storani, Carlotta Malagoli, Tommaso Filippini, Marco Tamelli, Marcella Malavolti, Fausto Nicolini, Marco Vinceti

**Affiliations:** 1 Department of Biomedical, Metabolic and Neural Sciences, CREAGEN—Environmental, Genetic and Nutritional Epidemiology Research Center, University of Modena and Reggio Emilia, Via Campi 287, 41125 Modena, Italy; lucia.borsari@unimore.it (L.B.); carlotta.malagoli@unimore.it (C.M.); tommaso.filippini@unimore.it (T.F.); marcella.malavolti@unimore.it (M.M.); 2 Clinical and Experimental Medicine PhD Program, University of Modena and Reggio Emilia, 41125 Modena, Italy; 3 Local Health Authority of Reggio Emilia-IRCCS, via Amendola 2, 42122 Reggio Emilia, Italy; simone.storani@ausl.re.it (S.S.); fausto.nicolini@ausl.re.it (F.N.); 4 Promotion Health Researcher, League against Cancer, via Amendola 2, 42122 Reggio Emilia, Italy; marco.tamelli@luoghidiprevenzione.it; 5 Department of Epidemiology, Boston University School of Public Health, Boston, MA 02118, USA

**Keywords:** tobacco smoking, smoking cessation, smoking cessation services, waiting times, referral

## Abstract

In Italy, the National Health Service offers specialized evidence-based support to smokers who would like to quit through smoking cessation (SC) services. We conducted a two-year prospective study, involving all 288 subjects treated for smoking cessation at the SC service of Reggio Emilia, to assess the association of referral sources and waiting times with the risk of treatment failure, by following participants up to one year after the quit date. We performed Cox-regression analysis, including demographic and smoking-related characteristics as confounding variables. The treatment failure rate at 12 months was 59.4% (171/288), including only 12 subjects lost to follow-up. The main mode of entry was self-referral (42.4%), followed by 32.6% from general practice, 17.4% from hospital and 7.6% from other sources. Only 27.8% participants were involved in the SC-program within 60 days of the first contact, as the guidelines suggest. The risk of treatment failure at 12 months showed little association with the type of referral source, while it correlated with waiting times ≥ 60 days (hazard ratio = 1.59; 95% confidence interval 1.10–2.29). This study provides evidence of long-term high quit rates from a SC service, with few subjects lost to follow-up and biochemical verification of almost all abstinent subjects. Timeliness in care provision could further improve the outcome.

## 1. Introduction

Tobacco use is the main preventable cause of death in the world, accounting for approximately 16% of all deaths in Europe [1]. Although the health risks related to smoking habits are currently well known, the prevalence rate remains dramatically high. In 2015, according to a survey conducted by Doxa (the Italian agency of the Worldwide Independent Network/Gallup International Association), 22% of the Italian adult population (>15 years) includes active smokers [2]. Several measures have been taken to control this smoking epidemic, such as anti-tobacco campaigns, tax increases on cigarettes, legal restrictions, tobacco package warning messages and specific interventions regarding smoking cessation, as suggested in the 2005 World Health Organization Framework Convention on Tobacco Control [3,4,5,6]. 

In Italy, the National Health Service supports smokers who would like to quit through smoking cessation (SC) services implementing individual or group interventions according to national and international guidelines [7,8,9,10], by using behavioral change techniques in combination with tailored pharmacotherapy [11]. A recent Cochrane review [12], suggested that group therapy provides better results in helping people stop smoking than self-help. There was, however, not enough evidence to evaluate whether groups are more effective or cost-effective than intensive individual counselling.

Several studies have demonstrated that socio-demographic characteristics and smoking behavior have a significant impact on smoking cessation. In particular, female gender, younger age [13,14,15], a lower education level or socioeconomic status [16,17,18], history of past or current psychiatric disorders [19,20], high level of nicotine dependence [21,22] and a lack of tobacco-related diseases [23] are associated with an increased risk of failure in quit attempts. In addition to smoker-related factors, SC service characteristics and organization may also influence smoking cessation. For example, timeliness in taking care of subjects who request tobacco-addiction treatment has been singled out [8,9,10,11], since smokers’ motivation to quit can change quickly, depending on the stage of change, as described by the so called “transtheoretical model” [24,25]. Nevertheless, the influence of waiting times during SC-programs and its impact on outcomes has been poorly evaluated to date. In addition, different types of referral sources to SC services have been assessed and showed contrasting results. For example, a higher cessation rate was found in self-referred subjects than for smokers referred from other sources [26]. In addition, another study highlighted better outcomes in subjects coming from general practitioners (GPs) than for self-referred subjects [27]. 

The aim of this study is to investigate whether referral sources and waiting times influence smoking cessation in an Italian SC service.

## 2. Methods

### 2.1. Research Setting 

The SC service of the Reggio Emilia province (around 500,000 inhabitants), in Northern Italy, is managed jointly by the National Health Service Local Health Authority and a not-for-profit organization, the Italian League against Cancer (LILT) [28]. This unit began to operate in early 2011. The permanent staff consists of one physician, two psychologists and three nurses. All staff members are specialists in smoking cessation techniques, thanks to their participation in specific courses certified by the regional health authority. 

### 2.2. Treatment Regimen

**Figure 1:**
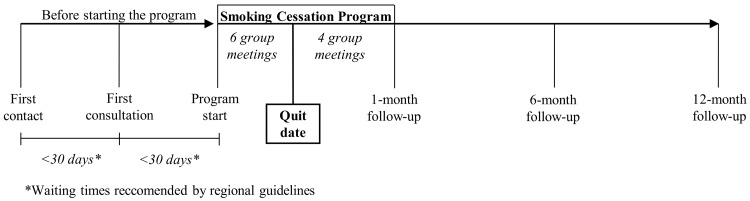
Steps towards stopping smoking at the smoking cessation service of Reggio Emilia (Northern Italy).

The steps towards stopping smoking for subjects accessing the service are presented in Figure 1. 

After a request for tobacco-dependence treatment (first contact) is received, a first consultation is scheduled to examine the smoker’s stage of change according to the “transtheoretical” method [24,25], and to evaluate whether the subject is ready to begin the SC program. As part of the standard protocol for all individuals admitted to the SC service, we measure the baseline carbon monoxide (CO) level using a breath test during this first consultation. Typically, the service provides group-based cognitive–behavioral treatment with pharmacological support as indicated, according to smoking cessation treatment guidelines, such as nicotine replacement therapy (NRT) and buproprion [29]. Each group is activated after receiving at least ten requests. As a result, waiting times are sometimes long. Individual counselling is provided only in specific situations, particularly in relation to psychiatric disorders. These individuals (only six during the study period) were excluded from the study. 

Group-based SC programs are inspired by the five-day plan described by McFarland et al. [30,31] and consist of 10 meetings. Each meeting lasts about 2 h and is managed by a multidisciplinary team, including a physician. During the intensive phase (three weeks), two meetings per week are scheduled, with the aim of increasing the smokers’ motivation to quit. Smokers are supported in the gradual reduction of cigarettes until they reach the quit date. Within the group, all participants agree on the date when they are due to stop smoking, which is usually at the end of the intensive phase of the program. Group support of each individual’s efforts is encouraged. The criterion to be included in the next phase of the group-based program is the goal to quit within seven days of the intended quit date. After the quit date, one group meeting per week for one month is scheduled to help subjects in abstinence, and with developing and actively rehearsing coping strategies. Subjects are followed-up approximately 1, 6 and 12 months after their quit date to determine whether they have successfully quit smoking. Subjects were considered abstinent at follow-up if CO values were ≤7 ppm, according to the Russell criteria for the definition of abstinence and relapse [32]. 

### 2.3. Participants 

**Figure 2:**
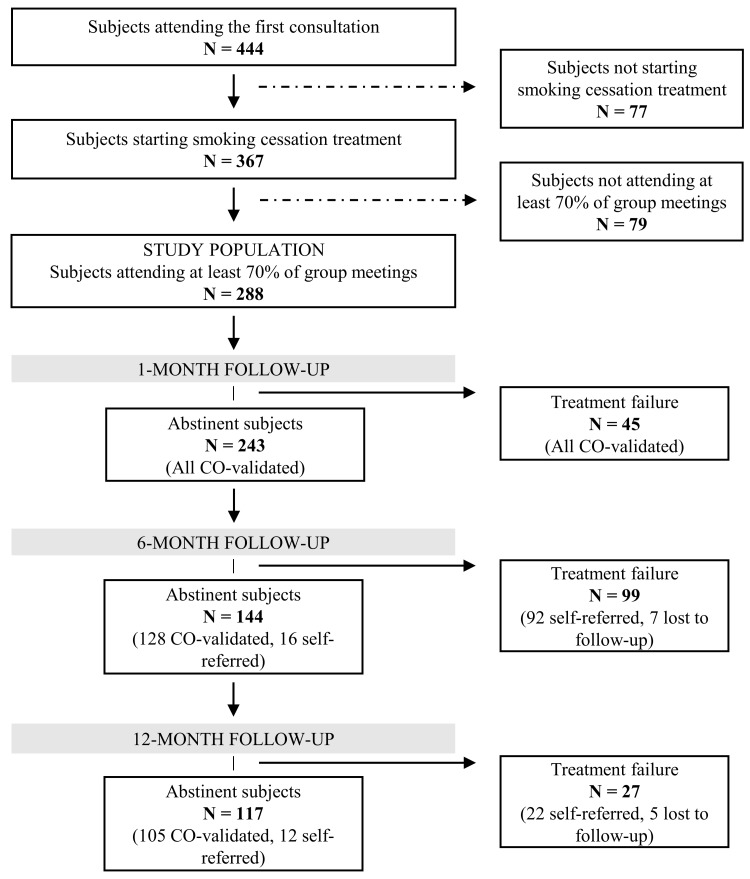
Flow chart of the study population.

We designed a prospective cohort study, including subjects treated for smoking cessation between June 2011 and May 2013. We considered in the analyses only subjects attending at least 70% of the group meetings (7/10 meetings). Of all the subjects (444) who had a first contact with the SC service to plan a smoking cessation program, 288 (64.9%) were included in the study (Figure 2). 

Smoking status was evaluated at 1, 6 and 12 months after the quit date. All the 288 subjects included in the study had CO-validated smoking status at the one-month follow-up. At the 6- and 12-month follow-ups, subjects not participating in the follow-up visits were contacted by phone. If phone counselling was not possible, we considered the subject as lost to follow-up. We applied an intention-to-treat approach, thus subjects who were lost to follow-up were counted as non-abstinent. All of the participants gave their written informed consent to be included in the study.

### 2.4. Collected Variables

We collected information about service-related characteristics, namely referral sources to SC services and waiting times. We categorized referral sources as follows: “self-referral”, “general practitioner/pharmacist”, “hospital” (i.e., subjects presenting as part of diagnostic and therapeutic care programs, usually following acute events), and “other sources” (i.e., subjects presenting after prevention interventions in workplaces). According to regional guidelines [8,9], waiting times between the first contact with the service and the beginning of the SC program should not exceed 60 days (i.e., 30 days between first contact and first consultation, and 30 days between first consultation and the beginning of the SC program).

During the first consultation, we also collected demographics (sex, age), clinical (actual mood or anxiety disorders requiring pharmacological treatment) and smoking-related variables, including age of onset, years as smokers and number of cigarettes smoked per day over the past 12 months. We administered the Fagerstrom test for nicotine dependence (FTND) to evaluate low (score < 6) and high (score ≥ 6) tobacco dependence [33]. 

### 2.5. Outcomes

The main aim of the study was to evaluate treatment failure rates in SC program participants at the SC service of Reggio Emilia. A secondary aim was to analyze whether waiting times and referral sources were associated with treatment failure. We evaluated treatment failure, defined as non-abstinence at 1, 6 and 12 months from the quit date. We considered the following as non-abstinence: CO-validated non-abstinence, self-reported non-abstinence and lost to follow-up. 

### 2.6. Data Analysis 

We analyzed the treatment failure rates according to participant and service-related characteristics. Then, we used survival analysis to evaluate the risk of smoking cessation treatment failure at the 12-month follow-up. We used log-rank analysis to compare the treatment failure Kaplan–Meier estimates across different groups, depending on the SC service referral source and the waiting time. Finally, we computed hazard ratios (HRs) and the 95% confidence interval (CI) of treatment failure at the 12-month follow-up in relation to the referral source and waiting time through the Cox proportional hazards regression model, adjusting the results according to the participants’ characteristics.

## 3. Results

The treatment failure rate at 12 months was 59.4%, meaning that 171/288 subjects were non-abstinent. We had only 12 subjects lost to follow-up. Most subjects underwent treatment failure within six months of the quit date (144/171, 84.2%), in particular in the period between one and six months (99/171, 57.9%). Only 15.8% of failures were between 6 and 12 months. Among subjects defined as abstinent at 12 months (117/288), we had biochemical verification for 89.7% (105/117). Treatment failure rates at 12 months according to the participant and service-related characteristics are shown in Table 1. 

The treatment failure rate was higher in women (61.3%) than in men (57.1%), in subjects aged <35 years (70.0%) than in the other age categories, and in subjects with anxiety disorders. As for smoking behavior, subjects who started smoking aged 15 or under and subjects with high nicotine dependence had higher treatment failure rates. Smokers for less than 20 years had a treatment failure rate of 78.6%, which was definitely higher compared to subjects who had been smokers for more than 20 years (57.3%), because that category included all subjects aged <35 years. 

**Figure 3:**
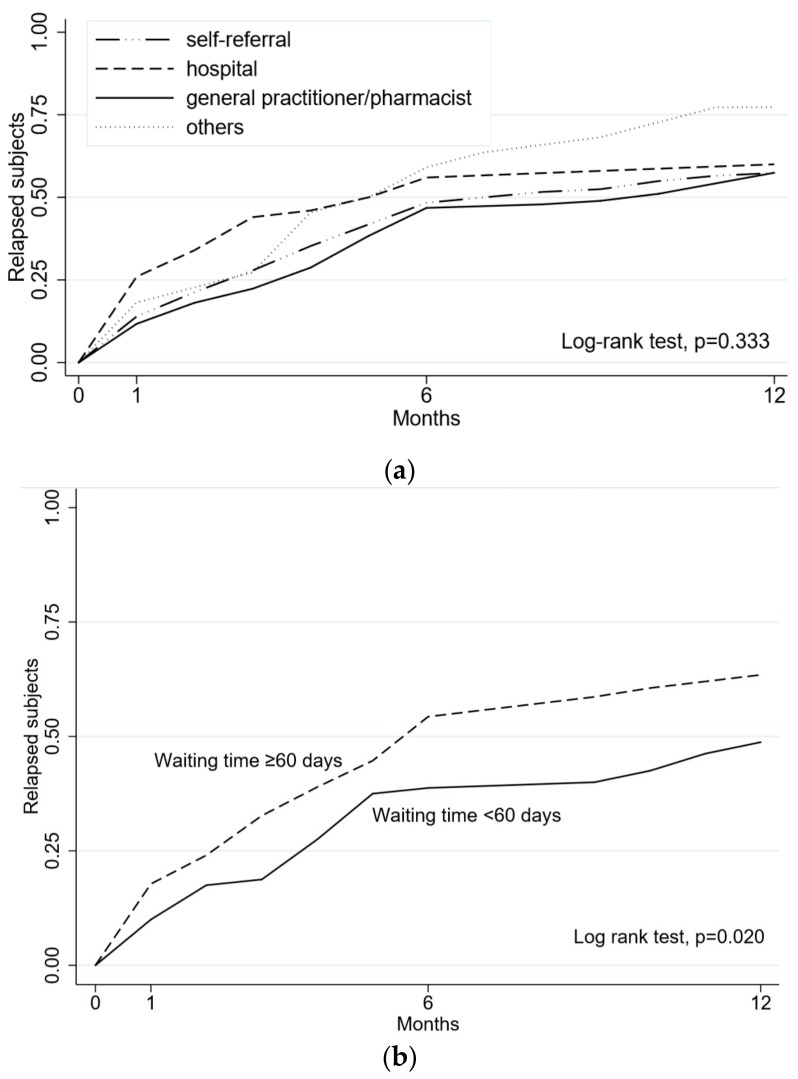
(**a**) Kaplan–Meier estimates of treatment failure at 12 months at the smoking cessation service of Reggio Emilia, according to the referral source. (**b**) Kaplan–Meier estimates of treatment failure at 12 months at the smoking cessation service of Reggio Emilia, according to waiting times from the request for smoking cessation and beginning of the smoking cessation program.

The majority of subjects (42.4%) had access to the SC service through self-referral, followed by 32.6% of subjects receiving GP/pharmacist advice, 17.4% referred from hospital and only 7.6% from other sources. These subjects were few in number, but they had the highest treatment failure rate at 12 months (77.3%). Participants referred by a GP/pharmacist failed in 60% of cases, those referred from a hospital failed in 57.5% of cases and self-referrals in 57.4% of cases. The Kaplan–Meier estimates and log-rank tests according to the referral source are presented in Figure 3a. Regarding waiting times, only 80 subjects (27.8%) were involved in the SC program within 60 days of the first contact, with a median waiting time of 81 days (range 2–218). Of these, 48.7% had treatment failure at 12 months, compared with 63.5% among subjects waiting ≥ 60 days. The Kaplan–Meier estimates and log-rank tests according to the waiting times are presented in Figure 3b.

Based on a multivariable Cox-regression analysis (Table 2), the risk of treatment failure at 12 months showed little association with the different types of referral source. On the other hand, treatment failure correlated with waiting times ≥ 60 days, with HR = 1.59 (95% CI: 1.10–2.29) in the fully-adjusted model for participant and smoking-related characteristics.

## 4. Discussion

This study provides evidence of the long-term treatment failure rates among participants in smoking cessation programs provided by an Italian smoking cessation service. Furthermore, the impact of the waiting times and referral sources on treatment failure was assessed. To the best of our knowledge, this is the first study evaluating waiting times as a contributory factor leading to failure in smoking cessation interventions. As far as referral sources are concerned, only results from studies performed in Canada and the United States are available [26,27]. 

The 12-month treatment failure rate of 59.4% is consistent with findings from a previous longitudinal study involving 41 Italian smoking cessation services [34]. In this study, Belleudi and colleagues compared the effectiveness of different smoking cessation treatments, finding that NRT combined with group therapy was the most effective therapeutic program. In this subgroup, the treatment failure at six months was 42.8%. International studies evaluating one-year outcomes reported generally higher relapse rates. For instance, studies providing brief advice from health personnel along with nicotine replacement therapy reported one-year relapse rates of 90–95% [35,36]. Conversely, other studies evaluating the effects of more intensive interventions (i.e., group-based/one-to-one cognitive–behavioral treatment with pharmacological support, similar to our study), typically documented a 70–85% relapse rate at one year [37,38,39,40]. A reason for our high success rate in long-term smoking cessation could be the type of support provided to smokers. We offer group-based treatments provided by a multidisciplinary team including smoking cessation specialists and physicians. In the United Kingdom, as in Italy, the National Health Service supports smokers who would like to quit through SC services, providing cognitive–behavioral treatment with pharmacological support. However, these services mainly offer one-to-one meetings with nurses, midwives or active members of the communities with little or no previous experience of health care intervention, as described by Judge et al. [41]. At the 52-week follow-up, they had a smoking cessation rate of 17.7%, including CO-validated and self-reported cases [42]. These services engage a larger number of smokers compared to the Reggio Emilia service. Nonetheless, the rate of subjects lost to one-year follow-up, which contributed to the treatment failure rate, is much higher (37.5% vs 4.2%). The high response rates for the Reggio Emilia SC service were reached through repeated attempts to contact smokers, which on the other hand could be very expensive and demanding in larger cohorts of subjects. In Denmark, in a study including 46,287 subjects treated by 423 SC services, Rasmussen et al. reported a quit rate of 24% at six months, which rose to 33% if subjects who were lost to follow-up (26% of the total sample) were excluded [43].

The treatment failure rates in our sample were not considerably different according to the demographic, clinical and smoking-related characteristics of subjects. However, failure rates were slightly higher for women, participants younger than 35 years, those affected by anxiety disorders, those starting to smoke before the age of 15, and those with high nicotine dependence, in accordance with the current literature [41,42,43]. The distribution of participants according to the referral source was similar to that of English SC services [41], with the majority of subjects being self-referred. 

Subjects referred from GPs/pharmacists or from “other sources” had slightly higher treatment failure rates than those from hospitals or who self-referred. However, after multivariable analysis the different types of referral source showed little association with risk of long-term treatment failure. The influence of referral sources on smoking cessation outcomes should be further investigated on larger samples of smokers participating in SC programs. In a study conducted on the Canadian population [26], the higher relapse risk found in subjects with referral sources other than hospitals or self-referral was explained through varying nicotine dependence levels across patient referral sources. The authors also found that engagers from general practice had a much lower realization of the importance of quitting compared to those who self-referred. This supports our initial hypothesis, that subjects coming from general practice could have a weaker and only transient motivation to quit, compared with subjects who decided independently or those referred from hospitals following an acute event. However, the evidence in the literature is poor and generally contrasting. In a study performed on data from the Arizona Smoker’s Helpline database, authors found that self-referral patients are less prone to quit than those referred by doctors or other health care providers [27], although a different type of smoking cessation intervention was administered. 

Waiting times were observed to have an impact on the risk of smoking treatment failure. In particular, subjects waiting >60 days before beginning SC programs proved to be about 1.5 times more likely to fail in smoking cessation at 12 months. The proportion of smokers waiting more than 60 days before inclusion in the SC program was very large in our sample (76.8%). Therefore, our results are important in order to improve the organization of SC services and to guarantee timeliness in the provision of health care, thus increasing success rates in smoking cessation interventions. In other clinical fields, waiting times already represent an important indicator to evaluate the appropriateness of the provided care and the impact on outcomes, such as increases in disease severity and disease-related morbidity [44,45,46,47], and the public’s confidence in the health care system [48]. Our results confirm that in cognitive–behavioral treatments, compliance with waiting times is also crucial to reaching the goal. In SC programs, the outcome is closely related to the stage of the subjects’ motivation, so that long waiting times between first contact and inclusion in the program could lead subjects to recede from the determinative to the contemplative phase [25]. 

Our study has some strengths compared with previous investigations. First of all, we performed a long-term evaluation with only 4.2% of participants lost at one-year. Furthermore, we had biochemical verification of smoking abstinence for almost all patients (89.8%), minimizing the possible overestimation of quit rates based on self-reported data. 

A few limitations should also be outlined. We enrolled a low number of subjects, compared with SC services serving a population similar to Reggio Emilia. For instance, the SC service of North Cumbria in England (around 325,000 inhabitants) enrolled 3825 subjects over a two-year period [41]. In this context, great efforts have been invested in smoker engagement through advisers who were active members in their local community. However, the response rates at follow-up are usually much lower in such large cohorts. 

## 5. Conclusions

The findings from this study may have implications for the future monitoring and evaluation of smoking cessation services. A large number of smokers relapsed at six months, as did a considerable amount of subjects between 6 and 12 months. Therefore, long-term follow-up is important to assess the contribution of services to reducing smoking prevalence. Long-term follow-up provides accurate results only if reasonable response rates are achieved. Thus, long-term investments and strategies to maintain contact with treated subjects should be implemented to minimize the likelihood of subjects being lost to follow-up. 

Service-related characteristics should be taken into account in the evaluation of factors affecting the outcomes when evaluating smoking cessation programs. In particular, our results regarding the relationship between waiting times and smoking cessation suggest that timeliness in smoking cessation care provision could definitely decrease the risk of treatment failure. Further studies, including larger samples of smokers, are needed to refine our understanding of the impact of waiting times and referral sources on smoking cessation treatment failure. 

## Figures and Tables

**Table 1:** Treatment failure rates at 12 months among subjects treated for smoking cessation at the smoking cessation service of Reggio Emilia between June 2011 and May 2013, according to participant and service-related characteristics.

Characteristics	N	%	Treatment Failure Rate at 12-Month (%)
*Participants’ characteristics*			
**Sex**			
Female	160	55.6	61.3
Male	128	44.4	57.0
**Age**			
<35 years	20	6.9	70.0
35–50 years	90	31.3	56.7
>50 years	178	61.8	59.6
**Mood and anxiety disorders**			
Yes	60	20.8	68.3
No	228	79.2	57.0
**Years as smoker**			
≤20 years	28	9.7	78.6
>20 years	260	90.3	57.3
**Age started regular smoking**			
≤15 years	108	37.5	63.0
>15 years	180	62.5	57.2
**Type of smoker over the past 12 months**			
Heavy (≥1 pack per day)	174	60.4	59.8
Light (<1 pack per day)	114	39.6	58.8
**Nicotine dependance**			
High-very high (FTND ≥ 6)	67	23.3	65.7
Low-medium (FTND < 6)	221	76.7	57.5
*Service-related characteristics*			
**Referral source**			
Self-referral	122	42.4	57.4
GP/Pharmacyst	94	32.6	60.0
Hospital	50	17.4	57.5
Other	22	7.6	77.3
**Waiting times before starting SC-program**			
≥60 days	208	72.2	63.5
<60 days	80	27.8	48.7
**Total**	**288**	**100**	**59.4**

Abbreviation: FTND = Fagerstrom test for nicotine dependence; GP = general practitioner.

**Table 2:** Risk of treatment failure at 12 months in relation to referral sources and waiting times at the smoking cessation service of Reggio Emilia, 2011–2013.

Service-Related Characteristics	Model 1 *		Model 2 ^#^	
	HR	95% CI	HR	95% CI
**Referral source**				
Self-referral	Reference		Reference	
General practitioner/pharmacist	1.02	0.71–1.46	1.04	0.73–1.50
Hospital	1.25	0.81–1.93	1.25	0.81–1.95
Other sources	1.51	0.88–2.57	1.47	0.85–2.52
**Waiting times before starting SC-programs**				
<60 days	Reference		Reference	
≥60 days	1.51	1.05–2.17	1.59	1.10–2.29

* Model 1: Cox regression model adjusted for sex, age and anxiety disorders. ^#^ Model 2: Cox regression model adjusted for sex, age and anxiety disorders, regular-smoking starting age, packs of cigarettes per day, nicotine dependence. HR: hazard ratio. CI: confidence Interval.

## Abbreviations

SC	smoking cessation
GP	general practitioner
CO	carbon monoxide
FTND	Fagerstrom test for nicotine dependence
RR	relative risk
CI	confidence Interval
HR	hazard ratio
